# The Relationship Between Urban Characteristics and Non-Communicable Diseases—Conceptual Framework of the HORUS Project

**DOI:** 10.3390/ijerph23060759

**Published:** 2026-06-05

**Authors:** Sven Maričić, Denis Juraga, Tomislav Rukavina, Darko Roviš, Zlatko Trobonjača, Mihaela Marinović Glavić, Lovorka Bilajac, Vanja Vasiljev

**Affiliations:** 1 Centre for Biomodelling and Innovations in Medicine, Faculty of Medicine, University of Rijeka, Braće Branchetta 20/1, 51000 Rijeka, Primorje-Gorski Kotar County, Croatia; sven.maricic@uniri.hr; 2 Faculty of Engineering, Juraj Dobrila University of Pula, Negrijeva 6, 52100 Pula, Istarska County, Croatia; 3 Department of Social Medicine and Epidemiology, Faculty of Medicine, University of Rijeka, Braće Branchetta 20/1, 51000 Rijeka, Primorje-Gorski Kotar County, Croatia; tomislav.rukavina@uniri.hr (T.R.); darko.rovis@uniri.hr (D.R.); mihaela.marinovic@uniri.hr (M.M.G.); lovorka.bilajac@uniri.hr (L.B.); 4 Department of Physiology, Immunology and Pathophysiology, Faculty of Medicine, University of Rijeka, 51000 Rijeka, Primorje-Gorski Kotar County, Croatia; zlatko.trobonjaca@uniri.hr

**Keywords:** computer models, city planning, geographic information systems, non-communicable chronic diseases, public health, urban health

## Abstract

**Highlights:**

**Public health relevance—How does this work relate to a public health issue?**

- Urban characteristics influence exposure to health-promoting or health-damaging conditions linked to non-communicable diseases.
- The HORUS project addresses public health by linking urban planning, behavioral risk factors, and spatial health inequalities.

**Public health significance—Why is this work of significance to public health?**

- The manuscript provides a conceptual framework for integrating urban mapping, GIS, and participatory methods in non-communicable disease prevention.
- It highlights how vulnerable urban populations may benefit from healthier built environments and evidence-informed planning.

**Public health implications—What are the key implications or messages for practitioners, policy makers and/or researchers in public health?**

- Policy makers and urban planners can use the proposed framework to identify health-related urban inequalities and design-targeted interventions.
- Researchers and public health practitioners may use GIS, participatory approaches, and citizen-generated data to support healthier urban environments.

**Abstract:**

The HORUS project investigates the interface between urban planning and public health, focusing on the reduction in non-communicable diseases through innovative urban planning and technological integration. Using geographic information systems, the project will develop advanced urban mapping and analysis tools to visualize and tackle health inequalities. The participatory approach of technologies will actively engage communities and empower citizens to shape a healthier urban environment. Through multidimensional methodology, including qualitative research and natural experiments, HORUS will align urban planning with public health needs. The project will target modifiable risk factors (physical inactivity, unhealthy diet and substance use) and will promote behavior change and environmental redesign to reduce the prevalence of non-communicable diseases. The integration of digital technologies will not only improve the assessment of urban health but also facilitate evidence-based interventions tailored to vulnerable populations. HORUS will provide practical applications for policy makers and urban planners by providing actionable frameworks for incorporating health-promoting features into urban design. This holistic approach will help create resilient cities that prioritize public health and shape the future urban environment. The project is an example of the transformative potential of aligning technology, policy and community engagement to effectively address the challenges of urbanization, and non-communicable diseases.

## 1. Introduction

Improving urban design is essential for population health as the urban environment directly impacts health outcomes. Evaluating how built environments promote active transportation requires shifting away from passive scoping reviews. Dynamic interventions are now assessed through advanced human–computer interaction models—specifically ‘walking the digital street’ protocols—which utilize advanced simulations to evaluate real-time human behavior and active urban mobility preferences directly prior to physical municipal execution [1]. Integrating green spaces and reducing urban densities, such as noise and traffic, can enhance mental well-being in cities that are well-planned. While traditional approaches emphasize localized neighborhood master plans to control noise and density factors, forward-looking public frameworks increasingly deploy multi-dimensional ‘digital twins’ [2]. Prioritizing the population health issues in urban architecture is essential for the efficient management of infectious diseases, as exemplified by the COVID-19 pandemic. Zhang and Li [3] noted that densely populated areas lacking proper planning experienced increased rates of the transmission of infective diseases. Healthy urban design promotes the attainment of sustainable development goals by advocating for social equity, guaranteeing access to essential services, healthy food, and active transportation options [4]. The elements have a crucial impact in diminishing health disparities. Moreover, cities that integrate health policies into their urban planning are better equipped to tackle the challenges presented by aging populations by enhancing accessibility and community support networks [5]. Hence, the existing evidence clearly suggests that integrating health promotion into urban design is a vital step towards enhancing public health, resilience, and general quality of life in urban populations.

Healthy cities in Europe exemplify a holistic approach to urban planning and the advancement of public health [1], aiming to create surroundings that foster well-being in various aspects of life. These cities prioritize activities aimed at boosting physical exercise, improving access to green spaces, encouraging sustainable transportation, and assuring equal access to healthcare services. According to a report on the quality of life in European cities [6], Zurich, Copenhagen, and Groningen are acknowledged for their superior quality of life, attributed to resident contentment, inclusion, and safety. They emphasize public health by advocating for physical exercise, facilitating access to green places, supporting sustainable transportation, and guaranteeing accessible healthcare services. These efforts jointly promote inhabitants’ well-being and improve the quality of life in these cities.

**Figure 1:**
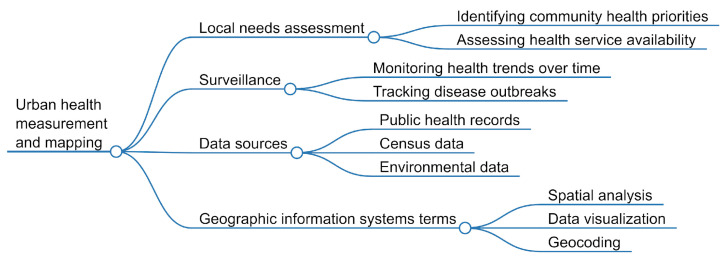
The main components of health assessment and mapping.

Urbanization has a significant impact on public health and presents both challenges and opportunities. Rather than simply viewing environmental risks through the lens of individual ‘urban-associated diseases’, contemporary public health demands a broader systemic evolution. As established by Hu et al. [7], spatial epidemiology is undergoing a definitive paradigm shift away from reactive infectious tracking toward the continuous monitoring of structural, macro-level chronic disease environments. Addressing these problems requires a comprehensive understanding of the social determinants of health and the use of modern technologies to improve health outcomes in urban populations [8]. Urban areas’ complexity determination involves the use of sophisticated technology for effective mapping and analysis, with computer-aided engineering (CAE) modeling and geographic information system (GIS) mapping being essential tools in this field. Utilizing GIS data and visualization technologies shows great potential for urban health mapping [9,10,11] by providing a detailed representation and study of urban environments as well as making the data more accessible and understandable to a broader audience. This approach helps improve comprehension of the several elements affecting population health indicators. By utilizing urban area mapping, we explore its ability to improve community health programs, enhance public health interventions [12], and offer valuable information on the health trends of urban populations. By combining GIS data with participatory mapping technology, different stakeholders including health professionals, policy makers, and community residents may get better insight and understanding of the important health-related features of metropolitan areas. This collaboration could lead to valuable information for stakeholders regarding urban health environments. Visualizing GIS and other data simplifies complex urban phenomena, allowing public health professionals and policy makers to make evidence-based decisions that improve educational programs and public health strategies [13,14,15,16]. This knowledge can then lead to the deployment of public health strategies that are more targeted and efficient. Some of the main components of urban health assessment and mapping are presented in Figure 1.

The significance of the convergence of urban mapping and health determinants has been investigated in this paper, with a particular focus on the power of contemporary technology and applications. The implementation of the HORUS project in this context could illustrate the potential of innovation to enhance urban environments and public health.

## 2. Urban Area Mapping

**Figure 2:**
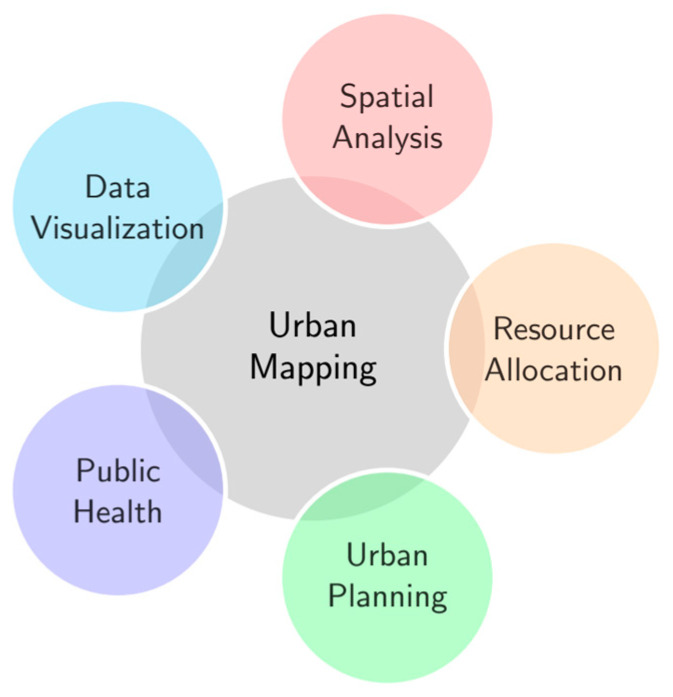
Urban mapping components.

The process of urban mapping, which is an important instrument in the field of public health, acts as an important link between urban planning and the outcomes of healthcare as well identifying present disparities [17,18,19]. Through the utilization of comprehensive geographical data, urban mapping provides professionals in the field of public health with the ability to visualize (Figure 2) the intricate relationship that exists between urban environments and health indicators. As highlighted by Favarão Leão et al. [9], achieving this visualization requires strict conceptual clarity between mere spatial inequalities in resource distribution and actual systemic spatial inequities to ensure that mapping translates into actionable urban planning policies.

This assists in gaining a more profound comprehension of the ways in which factors such as population density, accessibility to healthcare facilities, green spaces availability, and air pollution levels have an impact on public health and overall health of the population [20,21,22,23,24]. It is possible to define regions of health inequalities and vulnerabilities within urban populations with the assistance of this spatial analysis, which facilitates the allocation of resources and the execution of treatments that are specifically targeted. Urban mapping may be significant in promoting physical exercise and improving mental well-being by providing information for projects that increase park accessibility. This is accomplished by identifying places that have restricted access to green spaces. Additionally, in the context of infectious disease outbreaks, urban mapping is crucial for tracking transmission pathways in real time. This spatial data enables rapid, highly efficient intervention planning, a capability clearly demonstrated during the COVID-19 pandemic [3,25,26,27,28]. Moving beyond traditional descriptive mapping, recent advancements are shifting toward Urban Generative Intelligence (UGI) platforms, which anchor large language model agents inside sophisticated city simulators and knowledge graphs to model complex, context-aware human behaviors and mobility dynamics under emergency scenarios [25]. This demonstrates the power of geography in affecting public health policies and practices by demonstrating how cities may become more resilient, egalitarian, and conducive to the health and well-being of their residents through the incorporation of urban mapping into public health measures. Urban spatial structures are not static, they undergo continuous physical, political, and demographic temporal shifts. The HORUS framework models this dynamism by configuring rolling multi-temporal geospatial data tracks, actively calculating temporal data decay rates to prevent legacy artifacts from confounding longitudinal epidemiological validity over decade-long observation thresholds.

### 2.1. GIS in Public Health

This methodological transition is formally codified in the World Health Organization’s institutional policy. The newly ratified GIS for Health Roadmap 2025–2030 explicitly positions georeferenced spatial tracking as a mandatory structural standard for mapping NCD distributions and identifying pockets of vulnerability across the European region [29,30,31,32,33,34]. Public health professionals can analyze how environmental variables, like accessibility to green spaces and air quality, affect the emergence of NCDs, like type II diabetes, cardiovascular diseases, and asthma with the use of GIS technology, which integrates urban mapping with health data. The robust analytical capacity of GIS makes it possible to precisely identify regions in urban settings where NCDs are disproportionately prevalent. This makes it possible to create and implement extremely targeted public health interventions for those who most need them. To identify patterns and trends in NCDs, GIS combines data on disease prevalence with information about the environment and infrastructure. This information may be used to build proactive health policies and interventions. Researchers, decision-makers, and urban planners may examine the intricate link between rapidly increasing urbanization and how it affects risk factors for NCDs with the use of GIS, an essential tool in this decision-making process [9,35,36]. By using large amounts of data on population density, demographic trends, and municipal infrastructure, stakeholders may develop and put into effect comprehensive strategies aimed at lowering the risks associated with NCDs. This entails creating healthier urban environments via promoting walking and bicycling, enhancing public spaces, and lowering air pollution.

**Figure 3:**
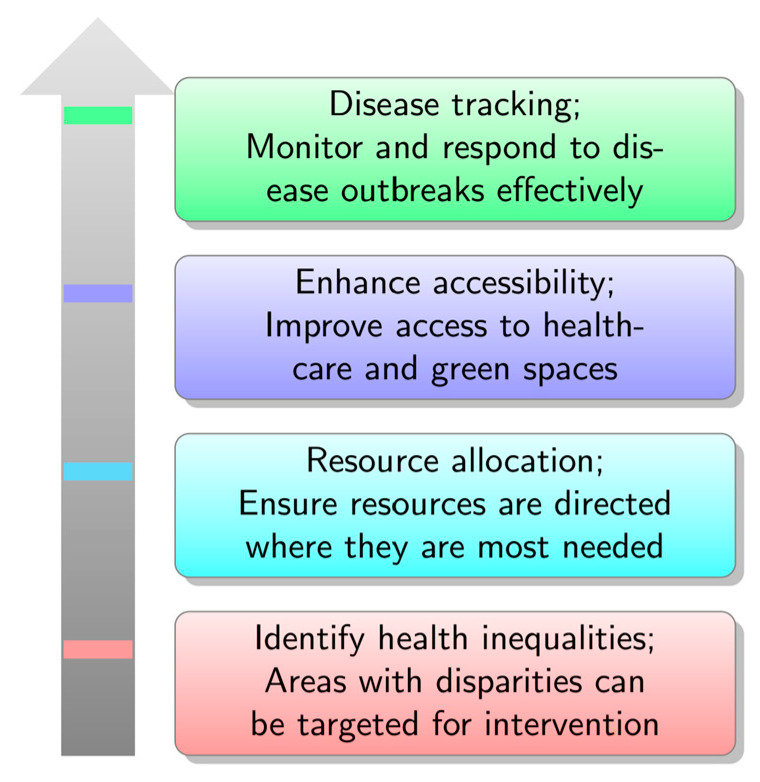
Benefits of using GIS in public health.

GIS-based impact assessment, as shown in Figure 3, of urban public health initiatives offers a versatile approach to healthcare management. It allows for ongoing adjustments and improvements to plans using comprehensive, region-specific data. This guarantees that treatments may successfully target the needs of urban populations while also being able to adjust to changes in the health environment. GIS plays a critical role in public health by advancing public health policies [29,34,37,38,39] in addressing NCDs in urban environment, enhancing evidence-based decision-making, and providing insight into the socio-environmental elements influencing health.

### 2.2. Computer-Aided Engineering (CAE), 3D Modeling and Virtual Reality (VR)

Computer-aided engineering (CAE) and Virtual Reality (VR) are crucial technologies that transform several sectors through the integration of simulation, visualization, and real-time interactions [40]. CAE use computer software to simulate performance, aiming to enhance product designs and address challenges in engineering across several sectors. This encompasses simulations in stress analysis, heat analysis, fluid flow analysis, and several other applications. Virtual reality, in contrast, generates an artificial environment that may closely resemble or diverge entirely from reality, enabling users to engage with a three-dimensional (3D) environment [40,41]. It has significant uses in training, education, design, and entertainment by offering immersive experiences.

**Figure 4:**
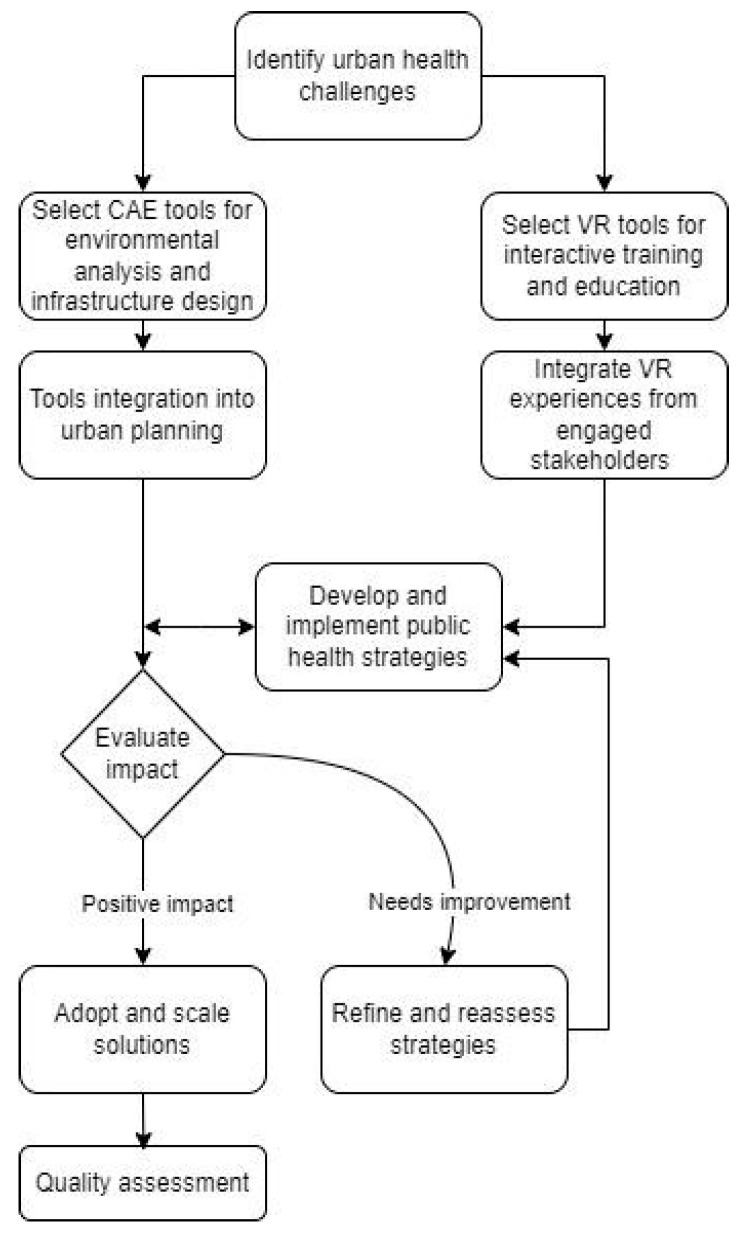
CAE and VR Integration in improving urban health planning.

The combination of CAE and VR technologies is of significance in the field of public health urban mapping. Rather than confining virtual reality strictly to clinical psychological diagnosis, contemporary frameworks apply VR to democratize urban architectural planning. By combining immersive environment engineering with real-time biometric eye-tracking, researchers can now isolate and quantify how restorative, biophilic urban modifications reduce ambient anxiety across varying socioeconomic demographics before breaking ground [42]. It has also proven to be a useful training tool for healthcare professionals [43]. The significance of these technologies in public health urban mapping is apparent as they provide thorough and interactive examination of urban surroundings, health facilities, and community requirements. VR can display intricate urban data, simulate hypothetical public health scenarios, and offer immersive experiences that assist in the process of planning and decision-making. CAE facilitates the examination and optimization of health facilities and infrastructure, guaranteeing their compliance with the most stringent safety and efficiency criteria (Figure 4).

Computer-aided engineering with its ability to simulate complex systems and VR with immersive visualization are transforming how we design and experience our cities (Figure 4). These technologies offer powerful tools for understanding and addressing public health challenges in urban environments. Urban design theories emphasize the crucial link between the built environment and public health [44]. By leveraging CAE and VR, planners can create “virtual cities” to test and optimize designs for walkability, access to green spaces, and social interaction—factors with proven impacts on community well-being. Their combination provides novel options for visualizing, evaluating, and optimizing urban health landscapes, eventually boosting community well-being and health outcomes. The use of these technologies in public health urban mapping is not only ground-breaking but also necessary for promoting more habitable, healthy, and environmentally friendly urban settings. Transitioning from traditional 2D overlays to 3D architectural modeling adds essential explanatory value by capturing environmental exposure verticality—such as localized atmospheric layer pooling, floor-level sound reverberation vectors, and localized shade indices within dense urban corridors. Where municipal 3D geometric coverage lacks complete city data files, the framework initiates iterative spatial imputation pipelines, blending satellite radar footprints with synthetic street profiles to patch coverage voids.

### 2.3. Artificial Intelligence (AI)

**Figure 5:**
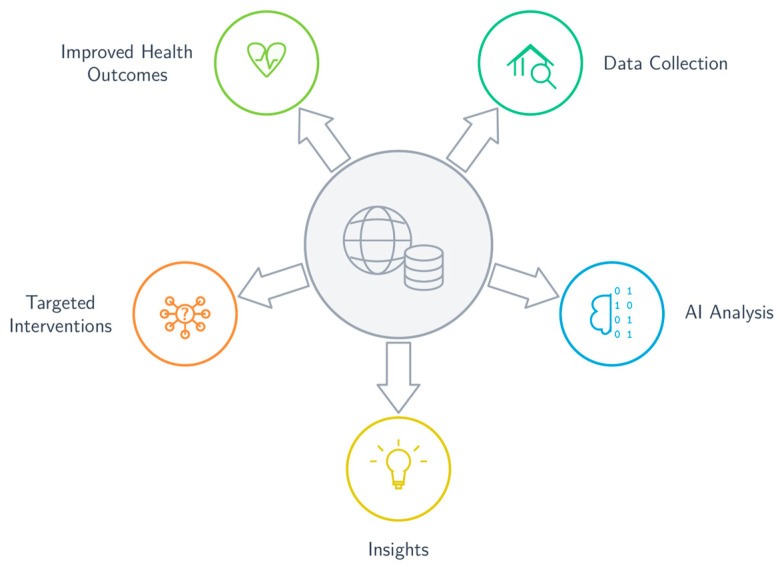
Implementation overview.

To model and analyze geographical data associated with disease outbreaks, health inequalities, and resource allocation, geographic data for public health employ large datasets and computer science [45]. The confluence of AI and public healthcare, which benefits both parties, is ushering in a new era in medical history. This innovative combination is expected to be a game-changer for the medical and healthcare domains. The use of AI into the mapping of metropolitan areas potentially signifies a crucial progression in public health research and intervention tactics. AI-powered analysis can provide in-depth information about the various elements [46] that contribute to health determinants, including environmental dangers and socioeconomic inequalities. To guarantee external validation generalizability across heterogeneous metropolitan environments, models incorporate standardized cross-geography domain transfer algorithms. Through the utilization of digital technology (Figure 5), such as machine learning algorithms, researchers and policy makers may efficiently evaluate and tackle the various factors that impact population health.

This technology allows for a detailed comprehension of preventative, clinical, and behavioral outcomes, therefore enabling precise treatments customized for specific communities. Implementing AI in urban area mapping may not only improve the accuracy of health interventions but also increase their scalability and effectiveness, eventually promoting better urban settings and enhancing population health outcomes.

## 3. Urbanization and Non-Communicable Diseases

Research on urbanization planning and its impact on NCDs is necessary to address the health challenges that are brought due to fast growing urban populations. Based on several research studies [47,48,49,50] this field has a significant relationship with the mapping of metropolitan areas. The importance of comprehending the influence that urbanization planning has on NCDs cannot be overstated. Through the process of mapping urban regions, this field of work attempts to address health concerns, especially unhealthy lifestyles and greater NCD rates that are brought by the rapidly expanding urban populations [51,52,53,54,55]. It has been demonstrated via research that urbanization has a significant influence on the epidemiology of NCDs, which include coronary heart disease, type II diabetes, and respiratory disorders. A considerable part of this impact may be linked to changes in lifestyle choices, such as nutrition, physical activity, and exposure to environmental toxins.

Urbanization results in a variety of health issues, such as the increase in NCDs. Urbanization, involving the movement of people from rural to urban regions, leads to lifestyle changes that make individuals more susceptible to it. This is further facilitated by metropolitan surroundings that frequently encourage physical inactivity, sedentary lives and unhealthy diets [56,57,58,59,60].

To identify areas that are at a higher risk of NCDs, Urban Area Mapping for Disease Surveillance requires the utilization of methods such as Bayesian hierarchical models [61,62,63]. This strategy is helpful in recognizing certain spatial patterns and hazard boundaries inside urban contexts, both of which are crucial for taking data-driven actions.

Public health professionals can identify hotspots and trends in NCDs in urban areas using GIS. Health data and urban mapping are used in GIS to examine, for instance, air quality and green space availability affect NCDs like asthma and cardiovascular diseases such as arterial hypertension and type II diabetes [64,65,66]. GIS-based research can identify communities disproportionately affected by NCDs to help design and implement targeted interventions in urban settings. GIS lets academics and policy makers examine how urbanization affects NCD risk factors by integrating data on urban infrastructure, population density, and health outcomes and devise strategies to mitigate them. It can also help evaluate urban public health initiatives for NCDs and improve them by allowing method modifications based on detailed geographical results.

## 4. The Implementation of the HORUS Project

Grounded in evidence-based interventions, HORUS is designed to support urban residents and, above all, those who are most vulnerable and socially disadvantaged, in taking up behaviors that lower their NCD risk. As the world continues to urbanize, the project positions itself as a driver of healthier urban settings and ways of living. Its central contribution is methodological: HORUS brings the urban and the behavioral together within a single epistemic and analytical framework rather than treating them as separate concerns. By equipping urban planners, policy makers and health professionals with practical instruments for confronting NCDs, the project intends to establish a reference point for urban health promotion and to help steer cities toward a healthier, more resilient, and more sustainable future.

Worldwide, NCDs such as type II diabetes and cardiovascular disease remain the foremost contributors to illness and death. Cardiovascular disease alone claims roughly 17.9 million lives each year, exceeding the toll of any other NCD, while type II diabetes is associated with around 2 million deaths annually [67,68]. The burden of these conditions is not distributed evenly: it falls disproportionately on disadvantaged groups, including low-income communities, migrants and ethnic minorities [69,70]. Several intertwined drivers explain this elevated prevalence, among them social and environmental conditions, ways of living, and behavioral determinants such as tobacco and alcohol consumption, poor diet and physical inactivity, together with aging, social exclusion, limited health literacy and restricted access to care [71]. For this reason, modifying individual-level factors, dietary patterns, physical activity and substance use, calls for behavior change interventions delivered in urban contexts, while environmental factors can be tackled by making better use of health-supportive urban built environments. Collectively, these considerations form the foundation of the HORUS project, which sets out to respond to the following gaps identified in the scientific literature.

Delivering effective responses to the NCD challenge requires the project to act on the underlying risk factors. The ‘4 × 4’ framework for NCDs [72] is instructive here, pairing the four principal NCDs, cardiovascular diseases, cancer, chronic respiratory diseases and type II diabetes, with four leading modifiable behaviors: tobacco and alcohol use, unhealthy diet, and physical inactivity. The framework holds that the majority of NCDs can be averted by acting on this small set of behaviors. Evidence indicates that behavior change techniques are the most effective means of reducing harmful alcohol consumption, smoking, poor dietary habits and inactivity [73,74,75,76,77]. Equally important, curbing the rise in NCDs depends on shaping environments that make healthy living easier. Urbanization stands out as a pivotal socio-environmental driver of this rise, since it limits contact with nature and health-promoting amenities while heightening exposure to climatic and environmental hazards. Much of the public health risk observed in cities arises precisely at the interface between how the physical-social environment functions and how people behave within it, that is, the individual health behaviors that a given setting encourages. The urban physical-social environment is recognized as a force shaping everyday choices: depending on personal characteristics, it can dispose some individuals toward health-risk behaviors and can likewise affect their subjective well-being.

Besides the conduction of qualitative public health methodology (focus groups and interviews) and the natural experiment, a research technique used in the social sciences to assess the causal effect of an intervention or public policy in the real world, the HORUS project will use GIS, allowing the qualitative results reported by the various included stakeholders who are directly or indirectly affected by the NCDs to be linked to a given geographic location and by the Healthy Cities Generator (HCG)-Citizens Module [78] that creates a visual, and scalable approach to collect citizens perception in relation to the use and design of built environment (this evaluation will also link the inputs of citizens with health impacts). The mix of qualitative methodologies with GIS and the HCG will ensure that social information is obtained by identifying the links between the psychosocial and functional characteristics of the urban environment and NCD risk behaviors, as well as the behavioral causal factors that drive the public health impacts of urban interventions, ultimately georeferencing this information by geographically organizing, analyzing and visualizing it through GIS. Thus, Public Participation in the field of GIS (PPSIG) has different implications and offers added benefits to the GIS approach. On the one hand, the PPSIG approach is more context- and problem-based than technology-based and focuses on community participation in the use of geographic information. Thus, the active participation of community members in decision-making processes that directly affect spatial planning is the undisputed key to the PPSIG approach [79]. In terms of added benefits, PPSIG has demonstrated potential to extend the use of spatial information to all relevant stakeholders, to obtain and manage people’s perceptions and conceptualizations of specific spatial contexts, to develop community awareness of specific local situations, and to strengthen community institutions as a promoter of people’s empowerment [79,80]. Given the importance of community involvement, the HORUS project is framed within the notion of the citizen science approach, which is defined as a collaborative approach to research that involves the public not only as research subjects or research advisors, but as direct collaborators in all aspects of the research process itself.

The decision to target these specific four non-communicable diseases is strategically driven by their shared reliance on modifiable built-environment risk behaviors, offering the highest systemic public health ROI for structural environmental redesigns. However, the operational manifestation of these risk behaviors is heavily stratified by neighborhood economic context. In low- and middle-income neighborhoods, the NCD burden is driven primarily by an absolute lack of infrastructure access and high exposure to uncontrolled industrial externalities; conversely, in high-income settings, chronic diseases track more closely with automated sedentary lifestyles and hyper-processed dietary access points, necessitating distinct urban intervention logic across varying socioeconomic thresholds.

## 5. Conclusions

This manuscript emphasizes the critical role of urban planning in addressing the rising burden of NCDs, a major global health challenge. The urban environment influences health outcomes by shaping behavior, exposure and access to health-promoting resources. The need for integrated and health-conscious urban planning has never been greater, especially as rapid urbanization continues to disproportionately affect vulnerable populations.

The HORUS project is a practical and forward-looking initiative to tackle these challenges. It provides a methodological framework that combines advanced technologies such as GIS and Healthy Cities Generator with community participation. Operationally, this closed-loop lifecycle synchronizes four distinct stages. These comprise GIS-driven baseline data ingestion via macro digital twins, automated predictive vulnerability mapping using multimodal GeoAI foundation models, community-led neighborhood co-design via immersive VR, and clinical validation against primary care digital health records. By mapping and analyzing the urban determinants of health, the project will provide actionable insights into the spatial and behavioral factors that contribute to NCDs. Importantly, the participatory approach will empower citizens and improve public health interventions by addressing both environmental and lifestyle determinants of health.

This work emphasizes the need to align public health and urban planning to mitigate the causes of NCDs and improve overall health equity. By translating research findings into policy-relevant tools and engaging multiple stakeholders, the HORUS project demonstrates how health-centered urban planning can contribute to healthier and more resilient cities. The results of the project will advocate for a shift in urban policy that prioritizes environments that promote well-being, sustainability and inclusion.

## Data Availability

No new data were created or analyzed in this study. Data sharing is not applicable to this article, as it is a conceptual framework review based on previously published sources, which are cited throughout and listed in the References.

## Abbreviations

The following abbreviations are used in this manuscript:

AI	Artificial Intelligence
CAE	Computer-Aided Engineering
GIS	Geographic Information Systems
HCG	Healthy Cities Generator
HORUS	Health Outcomes from Raised Urban Settings
NCDs	Non-Communicable Diseases
PPSIG	Public Participation in Geographic Information Systems
UGI	Urban Generative Intelligence
VR	Virtual Reality
